# Geology Can Drive the Diversity–Ecosystem Functioning Relationship in River Benthic Diatoms by Selecting for Species Functional Traits

**DOI:** 10.3390/biology12010081

**Published:** 2023-01-04

**Authors:** Evangelia Smeti, George Tsirtsis, Nikolaos Theodor Skoulikidis

**Affiliations:** 1 Institute of Marine Biological Resources and Inland Waters, Hellenic Centre for Marine Research, 46.7 km Athinon-Souniou Ave., 19013 Anavyssos, Greece; 2 Department of Marine Sciences, University of the Aegean, University Hill, 81100 Mytilene, Greece

**Keywords:** biofilm, diatoms, rivers, Greece, model simulations

## Abstract

**Simple Summary:**

The way that diversity affects ecosystem functioning is of great importance, as it helps us understand the health state of an ecosystem. Primary producers contribute to ecosystem functioning through biomass production, which is considered to be a proxy of ecosystem functioning. In rivers, the primary producers of the biofilm are diatoms, unicellular algae with cell walls of silica. In this study, we tested the way diatom species affect biomass production across nine rivers in Greece. Nutrient concentrations that drive primary production are linked to river geology. We found that the geological substrate of a river could be responsible for the diversity–biomass relationship: in rivers with a siliceous substrate, more diatom species increased biomass, whereas in rivers with a calcareous substrate, a change in diatom species number did not change biomass. By using model simulations, we found that this difference could be attributed to the different stages of the biofilm in time. Our results show the importance of different factors that affect diatom species, their functional traits and biomass production and what we should consider when testing for ecosystem functioning.

**Abstract:**

The biodiversity–ecosystem functioning (BEF) relationship has been studied extensively for the past 30 years, mainly in terrestrial plant ecosystems using experimental approaches. Field studies in aquatic systems are scarce, and considering primary producers, they mainly focus on phytoplankton assemblages, whereas benthic diatoms in rivers are considerably understudied in this regard. We performed a field study across nine rivers in Greece, and we coupled the observed field results with model simulations. We tested the hypothesis that the diversity–biomass (as a surrogate of ecosystem functioning) relationship in benthic diatoms would be affected by abiotic factors and would be time-dependent due to the highly dynamic nature of rivers. Indeed, geology played an important role in the form of the BEF relationship that was positive in siliceous and absent in calcareous substrates. Geology was responsible for nutrient concentrations, which, in turn, were responsible for the dominance of specific functional traits. Furthermore, model simulations showed the time dependence of the BEF form, as less mature assemblages tend to present a positive BEF. This was the first large-scale field study on the BEF relationship of benthic diatom assemblages, offering useful insights into the function and diversity of these overlooked ecosystems and assemblages.

## 1. Introduction

Ecosystem functioning comprises multiple processes that account for ecosystem health and sustain ecosystem services. For the past 30 years, research has been focusing on proving the pivotal role of diversity in driving ecosystem functioning [1]. In particular, the study of the form of the biodiversity–ecosystem functioning (BEF) relationship is considered important in light of global change and species extinctions [2]. It could be further used as a proxy of ecosystem health, resilience and species interactions, providing great insight into an ecosystem’s need for conservation [3,4]. BEF studies, especially early ones, have focused on experimental work, mainly on terrestrial plants, whereas aquatic environments and especially freshwater, remain understudied [5]. Field studies are rare, and regarding microalgae, they have focused on phytoplankton in Scandinavian and USA lakes [6,7] and the Baltic Sea [6,8]. A few studies on biofilm are limited to estuaries [9,10], but studies on the productivity of the river biofilm, especially on benthic diatoms, are almost missing (see [11]).

Diatoms, a major component of phytobenthos in rivers and the most diverse group of protists, are unicellular algae with silica cell walls, responsible for 20% of O_2_ production, and are important indicators of water quality [12]. As primary producers, their growth depends on nutrient concentrations and light, contributing immensely to primary biofilm productivity, an important ecosystem function. The importance of benthic diatoms on biofilm biomass production, along with their high diversity, renders them an ideal group of organisms for studying the BEF relationship in river biofilms. Furthermore, in recent years, functional traits related to cell size, to adherence to substrates and life forms are increasingly used in describing benthic diatom assemblages [13,14]. Despite the growing evidence that functional richness could be more important in driving ecosystem functions than taxonomic richness [15], functional diversity metrics are not widely used in BEF studies.

Although initial research focused on positive BEF relationships (i.e., an increase in ecosystem functions with increasing diversity), recent research and meta-analysis suggest different relationships (e.g., negative or hump-shaped relationships), depending on the type and duration of the study (i.e., observational field or experiment), the ecosystem type and the taxa studied [5]. Furthermore, the form of the BEF relationship can be affected by multiple diversity levels in space and time, such as the regional and local species pools or the initial and realized diversity [16]. Although they have the advantage of the large scale and the natural world, observational field studies can give ambiguous results. Natural systems are very complex and dynamic, and patterns could be masked by different diversity scales that are part of different assembly phases. On the other hand, modeling studies could provide mechanistic interpretations of the form of the BEF relationship [17,18]. For example, modeling studies on phytoplankton have suggested that when species utilize all the available resource space (e.g., when the system is at a mature steady-state), there is no BEF relationship, whereas when a part of the resource space stays unutilized (e.g., after a species extinction), then a strong positive BEF is apparent [17]. Therefore, coupling field observations with numerical modeling could give better insights into the drivers of the BEF relationship. 

The aim of this study was to test the form of the BEF relationship in benthic diatoms in rivers and the drivers of this relationship. Toward this aim, we collected samples along nine Greek rivers, varying in their geographical location, geology, drainage area and nutrient concentrations. As rivers are highly dynamic ecosystems and Greece is a country with diverse landscapes and geology, we hypothesized that a general BEF relationship would be hard to observe and that it would be driven by additional, possibly abiotic, factors. We further investigated species traits (size, attachment to the substrate) that can be responsible for the observed relationship. To further understand species coexistence and the consecutive biomass production during a succession of the biofilm in time, we ran model simulations and checked the BEF relationship at different maturity levels of the biofilm. We hypothesized that a less mature biofilm (at the beginning of time succession) would present a positive BEF, as resources are still unutilized, whereas a mature biofilm, where all available resources are used, would not present a BEF. To the best of our knowledge, this is the first large-scale observational study of the BEF relationship in benthic river diatoms, and although incomplete, it can give important insights into the function and diversity of these overlooked systems.

## 2. Materials and Methods

This study combined field observations with numerical modeling. Field sampling was conducted in river biofilm, comprising samples for both microscopic observation and biomass. Water physico-chemical parameters and nutrient concentrations were measured at each site. Diversity (i.e., species richness and evenness) of diatoms in the biofilm was defined using microscopic counts of species abundances, and functional traits were assigned to species to account for functional diversity. Biomass was measured in the lab as chlorophyll a concentration and was used as a proxy of ecosystem functioning. The shape of the BEF relationship was tested at different spatial scales, and nutrient concentrations and functional traits were investigated as possible drivers of the observed shape. Model simulations, using a well-known numerical model on species competition for available resources, were run, and the BEF relationship was observed at different time points to test for the dependence of the relationship to the maturity of the assemblage. 

### 2.1. Field Sampling

**Figure A1:**
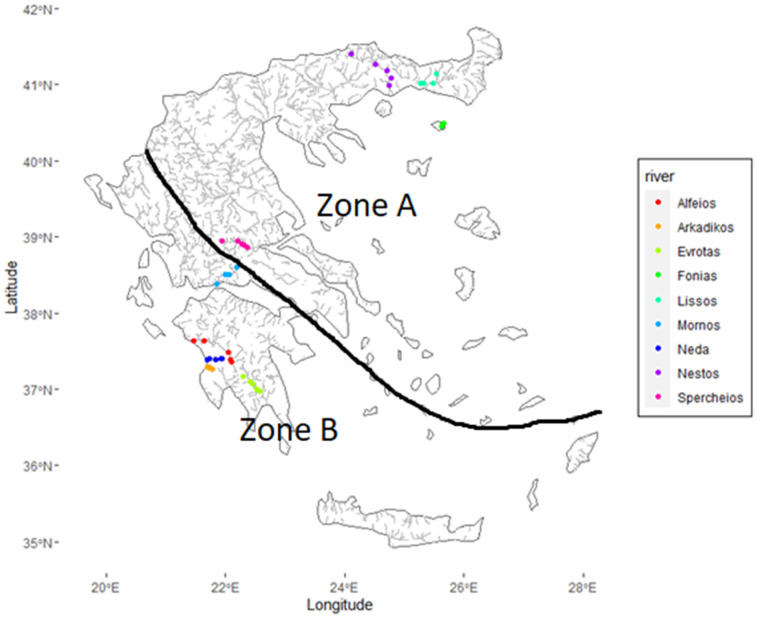
Map with the hydrographic network of Greece and the sampling points for the nine rivers. Hydrographic network is from https://geodata.gov.gr/en/dataset/udrographiko-diktuo (accessed on 6 December 2022). Map was created in R (v. 4.0.3), using packages rnaturalearth (v. 0.1.0), sf (v. 1.0-6) and ggplot2 (v. 3.5.3).

Nine Greek rivers (Nestos, Lissos, Fonias, Spercheios, Mornos, Alfeios, Arkadikos, Neda and Evrotas) were sampled in the summer of 2020, at a low flow period, when no major disturbances would cause shifts in the assemblages and their biomass (Figure A1, Table A1). These rivers were selected based on accessibility and appropriate sampling substrate (stones) as well as due to their differences in terms of size, geology and environmental conditions. In each river, five sampling sites were sampled from upstream to downstream, apart from Arkadikos and Lissos, where only four samples were taken. In order to ensure replication, in each site, three spots were sampled, each comprising three stones. From each stone, two surfaces of the defined area were scraped, the first used for chlorophyll analysis (immediately put in a dark bag and frozen) and the other for species identification and counting (preserved with 70% ethanol). This ensured the direct comparison between species diversity and biomass production. At each site, physico-chemical parameters (Temperature, DO, pH, Conductivity, Turbidity) were also measured in situ using a Portable multiparameter Aquaprobe, and water samples were collected for the determination of nutrients (NO_3_, NO_2_, NH_4_, TN, PO_4_, TP and SiO_2_). 

### 2.2. Analysis of Samples

In the laboratory, after filtration through 0.45 μm pore size membrane filters, nutrients were determined by a Skalar San++ Continuous Flow Analyzer [19]. For the determination of chlorophyll, the trichromatic equations were applied [20], where all three main chlorophylls were measured (Chl-a, Chl-b, Chl-c), and their concentrations were in mg/cm^2^. Chl-a is a measure of the whole phytobenthos biomass production, whereas Chl-c is more indicative of the biomass produced by benthic diatoms. 

Diatom species samples were treated with hot hydrogen peroxide to remove organic matter and obtain clean frustules, to be used for diatom species identification [21]. Clean frustules were mounted with Naphrax^®^, identified to species level with a light microscope (Nikon Eclipse Ci-L, Nikon Microscope Solutions, Europe) at 1000× magnification and counted until no more new species were detected in each sample. As the surface scraped out of the stone was defined, and the volume at each step of the procedure was also measured, the counting reflected the absolute abundance of cells per cm^2^. For the taxonomy, the work of [22] was mainly used. 

### 2.3. Data Analysis

**Figure A2:**
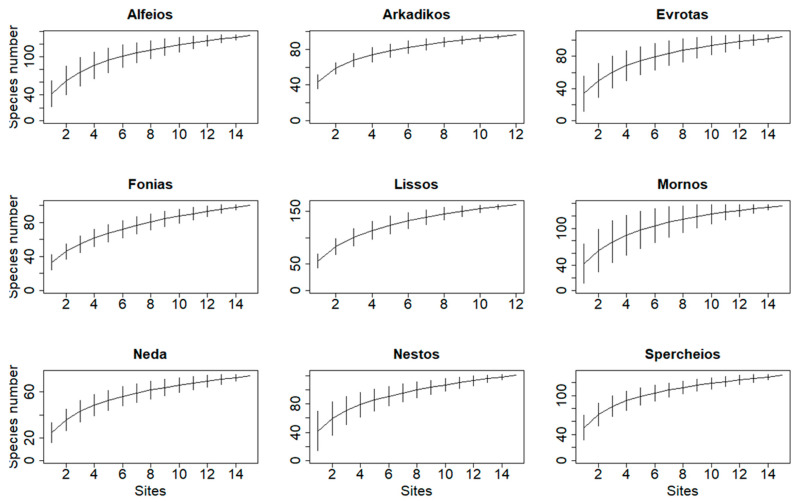
Species–accumulation plots for each river. When the curve reaches a plateau, we assume that sampling effort was enough to detect most of the species present. Analysis was performed in R (v. 4.0.3), using package vegan (v. 2.5-7).

**Figure A3:**
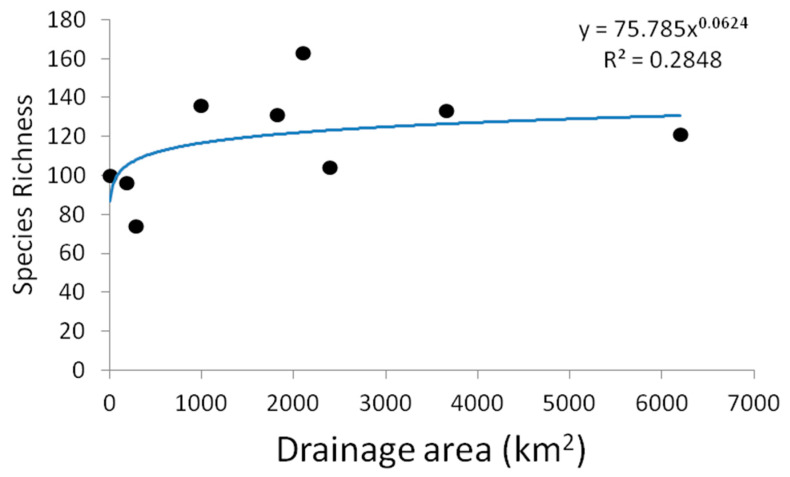
Species–area relationship for the sampled rivers. There is no clear evidence that species richness increases with increased drainage area.

In order to ensure that the sampling effort was adequate for all rivers examined, species accumulation curves (SACs) were constructed, showing that, indeed, most species were observed under the specific sampling and analysis procedures (Figure A2). Furthermore, to check that species richness counts were not biased due to macroecological patterns and large differences in the drainage areas, species–area relationships (SAR) for the selected rivers were performed, demonstrating the absence of a relationship between the area and the observed species richness (Figure A3). Another potentially confounding factor in natural systems is pollution. In the present study, pollution levels slightly differed, even between sites of the same river, based on a biological quality diatom index, but quality classes did not play an important role in the BEF relationship (interaction term *p*-value = 0.07). 

Taxonomic diversity was calculated using both species richness (S) and evenness (J), to account for the abundance distribution of individuals among species (i.e., assemblage structure). For calculating functional diversity, the functional richness index was used, defined as the total branch length of a functional dendrogram based on species’ functional traits [23]. Functional traits used were cell size (L/W ratio, biovolume), substrate adherence (high profile, low profile, motile and planktonic guilds), life forms (colonial, singular) and nitrogen fixation [14]. For the calculation of biovolume, equations of geometric shapes were used [24], and dimensions that could not be measured in our samples (e.g., cell height) were defined based on the literature [14]. The total biovolume of each sample was divided by the total abundance of the sample for the calculation of the average cell size of each assemblage, aiming to compare cell size between different groups of rivers [25].

Biomass metrics tested were Chl-a, expressing biomass production of the entire biofilm, Chl-c and Total biovolume, linked to benthic diatoms. Chl-a and Chl-c were highly correlated (Spearman r = 0.86, *p*-value < 0.001) and presented the same trends. Therefore, only Chl-a was used as a surrogate of biomass production. In order to show more clearly linear trends, Chl-a concentrations were ln-transformed.

The form of the relationship between the different diversity metrics and Chl-a was determined for the whole dataset, searching for a general pattern in the examined Greek rivers, as well as for each river separately to test for possible differences in the BEF relationship between rivers. Rivers were further grouped in two previously defined hydrochemical zones in Greece [26] with distinct silicate and phosphorus concentration ranges, affected by geology; in zone A, siliceous substrates are more prominent, and silicate and phosphorus concentrations in water are higher, whereas, in zone B, calcareous substrates dominate and silicate and phosphorus concentrations in water are lower, the latter due to adsorption on carbonate-rich particles and sediments [27,28]. Substrate geology (i.e., siliceous vs. calcareous) is known to select for species diatom species [22]. Therefore, as phosphorus and silica are important nutrients for diatom growth, their different concentrations in these two zones could affect assemblage characteristics and, thus, the corresponding BEF relationships. For the rest of the manuscript, when we refer to substrates (siliceous or calcareous), we refer to the geologic substrate of a river basin.

For testing the significance of the BEF relationship when used in different groups in the dataset, generalized linear mixed-effects models were used, with the river as a random factor. Data analyses and illustrations were performed in R (v. 4.0.3) [29], using packages vegan v. 2.5-7 [30], BAT v. 2.7.1 [31], lme4 v. 1.1-28 [32], ggplot2 v. 3.3.5 [33] and plotly 4.10.1 [34].

### 2.4. Model Simulations

Model simulations were performed in an effort to understand the importance of temporal succession and the maturity of the biofilm on the BEF relationship. Applied models were based on well-known models for phytoplankton competition for resources, assuming a continuous inflow of nutrients [35]. This model describes the population dynamics of 400 diatom species (N_i_) competing for two nutrients (*R_j_*), namely nitrogen and phosphorus. The initial species number (*n* = 400) is based on the total diatom species observed in all field samples.

(1) $$\frac{dN_{i}}{dt}=N_{i}\left(min\left(\frac{{\mu_{max}}_{i}\times R_{1}}{K_{1i}+R_{1}},\frac{{\mu_{max}}_{i}\times R_{2}}{K_{2i}+R_{2}}\right)-m_{i}\right),\;i=1-n$$

(2) $$\frac{dR_{i}}{dt}=D\left(S_{j}-R_{j}\right)-\overset{n}{\underset{i=1}{\sum }}c_{ji}\times min\left(\frac{{\mu_{max}}_{i}\times R_{1}}{K_{1i}+R_{1}},\frac{{\mu_{max}}_{i}\times R_{2}}{K_{2i}+R_{2}}\right)N_{i},\;j=1,2$$

*N_i_* is biomass of species i, and *R_j_* is the concentration of nutrient j; ${\mu_{max}}_{i}$ is the specific maximum growth rate of species i, and K_ji_ is the half-saturation constant of resource j for species i, based on the Monod model of growth limitation; m_i_ is the mortality induced by flushing, and it was calculated as the flushing rate (D) divided by the maximum growth rate of each species in the model; *D* is the nutrient flushing rate; *S_j_* is the input nutrient j concentration; and *c_ji_* is the intracellular content of nutrient j in species i. In diatoms, the maximum growth rate is linked to species size, with smaller species presenting a higher growth rate [36]. As larger species in a biofilm tend to be more affected by flushing than smaller species that tend to adhere to the substrate stronger, we assumed that they are more affected by flushing, which increases larger species mortality. 

The two nutrients used in the model are phosphorus and nitrogen, as they are both essential nutrients for growth. Based on field observations, phosphorus was mainly the limiting nutrient, whereas nitrogen limitation was also observed in some cases. The two nutrients in the model are added synchronously and in a continuous manner during the simulations at concentrations following the Redfield ratio. This synchronous and continuous flow simulates an ideal river environment, from upstream (nutrients entering the system) to downstream (nutrients flushing). Following the N:P:Si ratio in field observations, Si was never found to be limiting; therefore, even though an important nutrient for diatom growth, it was not considered in model simulations. 

Life history traits were assigned to species based on the literature values and on species functional traits that we observed in the field samples. The three main life history traits we focused on were the specific maximum growth rate (μ_max_), the competitive ability for Phosphorus (K_P_) and the competitive ability for Nitrogen (K_N_). Based on field data, smaller species tended to be at low nutrient concentrations; therefore, we assigned three groups of species, with each group being superior for two life history traits: one group consisted of fast-growing species with the increased competitive ability for phosphorus but not for nitrogen (high μ_max_ and low K_P_ but high K_N_), one group consisted of fast-growing species with the increased competitive ability for nitrogen (high μ_max_ and low K_N_ but high K_P_) and one group consisted of slow-growing species with the increased competitive ability both for phosphorus and nitrogen (low μ_max_ and low K_P_ and K_N_). Keeping a trade-off was important as the presence of a “superspecies”, superior for all traits, would exclude all other species, and thus, species richness in an assemblage would be extremely low. When assigning traits to virtual species, we made sure that there was a trade-off between R*_P_ and R*_N_, with a level of complementarity equal to 0.49 [37]. R* is the minimum concentration of a resource at which a species could keep its population stable, and it is a summary value of both growth rate and K_j_. Life history traits were assigned to species using R (v. 4.0.3).

The mathematical equations were solved numerically using a specially developed Fortran code following [37] and adapted to meet the characteristics of the studied systems. The BEF relationship was tested at each time step using the species richness and evenness against the log-transformed abundance of the 100 replicates. For each replicate, the initial biomass of each species and the total initial abundance varied randomly. The model parameters values, ranges and initial conditions are detailed in Table A2. 

## 3. Results

### 3.1. Field Observations

**Figure 1:**
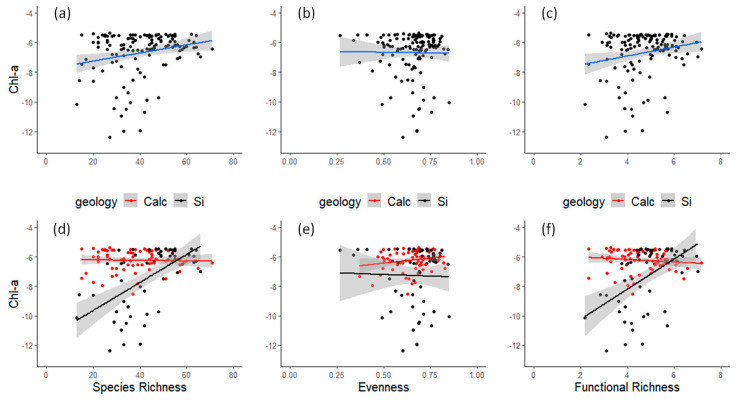
Diversity (expressed as (**a**,**d**) species richness S, (**b**,**e**) evenness J and (**c**,**f**) functional richness) and ecosystem functioning (presented as ln(Chl-a) on y-axis) relationships in each river on the whole dataset (**a**–**c**) and at different geological substrate levels (**d**–**f**).

The general BEF relationship (when all samples were pooled together) when using species richness (S) as the diversity predictor of biomass was positive, albeit rather weak (*p*-value < 0.01, Figure 1a). A seemingly similar but not significant trend was apparent when functional richness was used as a biomass predictor (*p*-value = 0.235, Figure 1c). The lack of a significant relationship was also present when evenness (J) was used as a diversity predictor of biomass (*p*-value = 0.676, Figure 1b).

**Figure A4:**
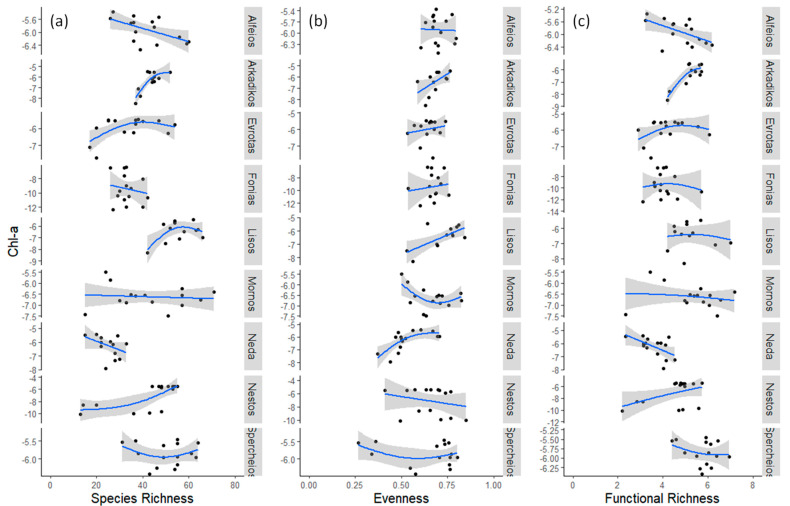
Diversity (expressed as (**a**) species richness-S, (**b**) evenness-J, (**c**) functional richness-FRichness in x-axis) and biomass (ln Chl-a in y-axis) relationship for each river.

**Figure A5:**
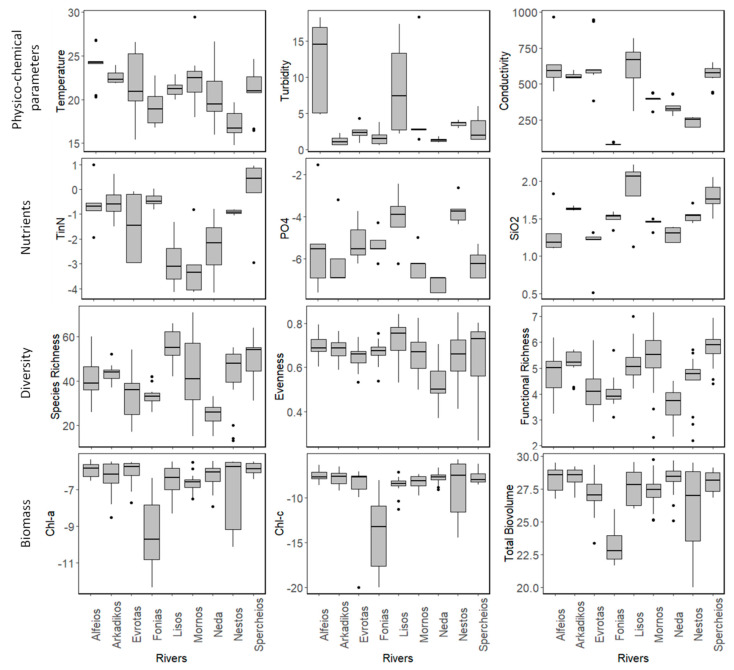
Physico-chemical (temperature, turbidity, conductivity), nutrient concentration (Si, TN, TP), diversity (species richness, functional richness, evenness) and biomass (Chl-a, Chla-c, Total biovolume) variation between the nine rivers of the study.

When each river was considered separately, the relationship between species richness and biomass production was variable between the different rivers sampled (Figure A4a). Indeed, Chl-a was best explained when the interaction between species richness and the river was also considered (adjusted R^2^ = 0.55, *p*-value < 0.001). This variation is also apparent when considering other diversity metrics (evenness J and functional richness, Figure A4b,c). Variability among rivers was also evident in environmental conditions, as depicted in the physico-chemical parameters and nutrient concentrations measured (Figure A5). 

A strong interaction effect is apparent when testing for the substrate geology (interaction term *p*-value < 0.001). In siliceous substrate, an increase in species richness resulted in an increase in biomass production (positive BEF-slope = 0.097, *p*-value < 0.001), whereas, in the calcareous substrate, an increase in species richness did not have any effect on biomass production (no BEF relationship-slope = −0.0014, *p*-value = 0.9—Figure 1d). Functional richness presented the same trend (interaction term *p*-value < 0.05, Figure 1f), but evenness (J) had no effect on predicting biomass ((interaction term *p*-value = 0.204, Figure 1e). There was no significant difference between species richness or biomass for the two groups of rivers.

**Figure 2:**
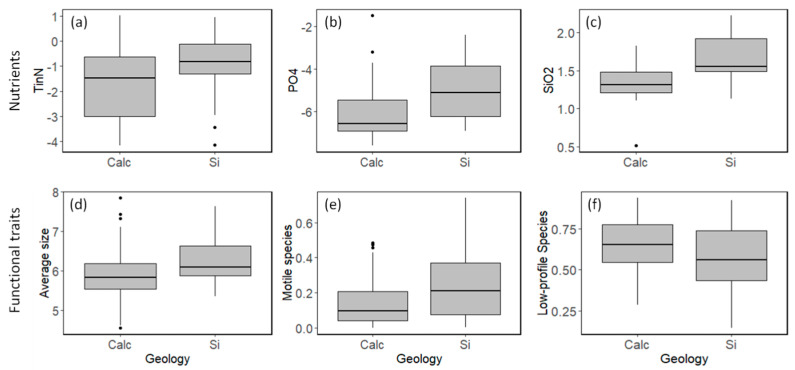
Difference between nutrient concentrations: (**a**) total inorganic nitrogen; (**b**) orthophosphates; (**c**) silicates and species functional traits; (**d**) average size in the assemblage; (**e**) relative abundance of motile species; (**f**) relative abundance of low-profile species between calcareous and siliceous substrates. Y-axis in (**a**–**d**) is ln-transformed.

**Figure 3:**
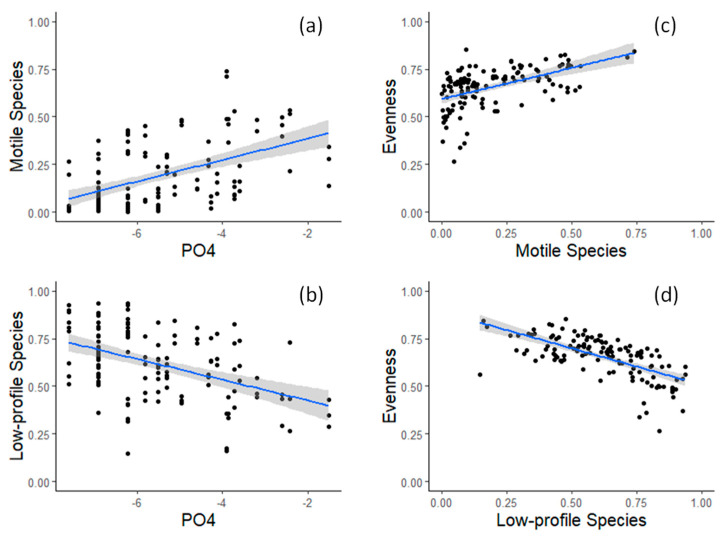
Relationship between PO4 concentration and diatom guilds: (**a**) relative abundance of motile species; (**b**) relative abundance of low profile species; and (**c**,**d**) the same diatom guilds and evenness J.

In rivers with a siliceous substrate, all tested nutrients (TinN (i.e., sum of NO_2_, NO_3_, NH_4_), PO_4_, SiO_2_) presented higher concentrations than in rivers with a calcareous substrate (*p*-value < 0.05–Figure 2a–c). Regarding species traits, rivers in siliceous substrates have diatom assemblages comprised of bigger and motile species, whereas rivers in calcareous substrates have diatom assemblages comprised of smaller, low-profile species (*p* < 0.05–Figure 2d–f). Overall, motile species tended to increase with increased phosphorus concentrations, whereas low-profile species tended to decrease with increased phosphorus concentrations (Figure 3a,b). The other guilds (high-profile and planktonic species) did not present any consistent relationship between geology or nutrient concentrations. Furthermore, a higher relative abundance of low-profile species resulted in high dominance assemblages (Figure 3d), whereas higher evenness was observed when more motile species were present (Figure 3c). 

### 3.2. Model Results

The above field results on nutrient concentrations and species guild and size indicate that small, fast-growing cells are also good competitors for phosphorus. This was the assumption we used in the model parameterization regarding life history traits of the initial species pool (explained in the methods above). 

**Figure 4:**
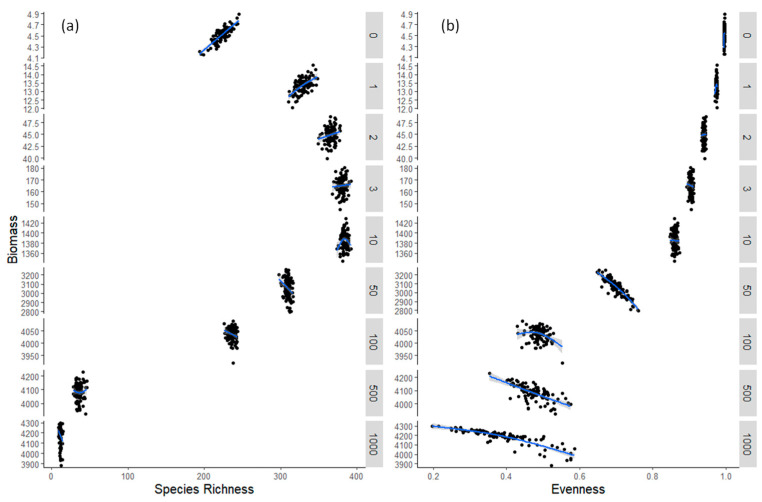
Model results between (**a**) species richness S and (**b**) evenness J (on x-axis) and biomass (y-axis) for different days (0–1000) during simulations. For each day, the 100 replicates are plotted.

Model results varied with time during the simulation. At the very beginning of the simulation period, species richness started to increase, along with total biomass, and the BEF relationship was positive for these initial time steps (Figure 4a, Table 1). During succession, once all the species presented detectable biomass, no significant relationship was apparent between species richness and total biomass production (Figure 4a, Table 1). Even later in succession, when species started to go extinct and the total biomass started to reach the maximum carrying capacity of the system, there was still no significant relationship, or a negative one, between species richness and total biomass production (Figure 4a, Table 1). However, species richness and total biomass did not vary a lot between replicates at later stages of succession. On the other hand, as species started to go extinct and the system reached its maximum biomass, evenness presented a higher variability between replicates and a negative relationship with total biomass, whereas assemblages with higher dominance also presented higher biomass (Figure 4b, Table 1).

**Figure 5:**
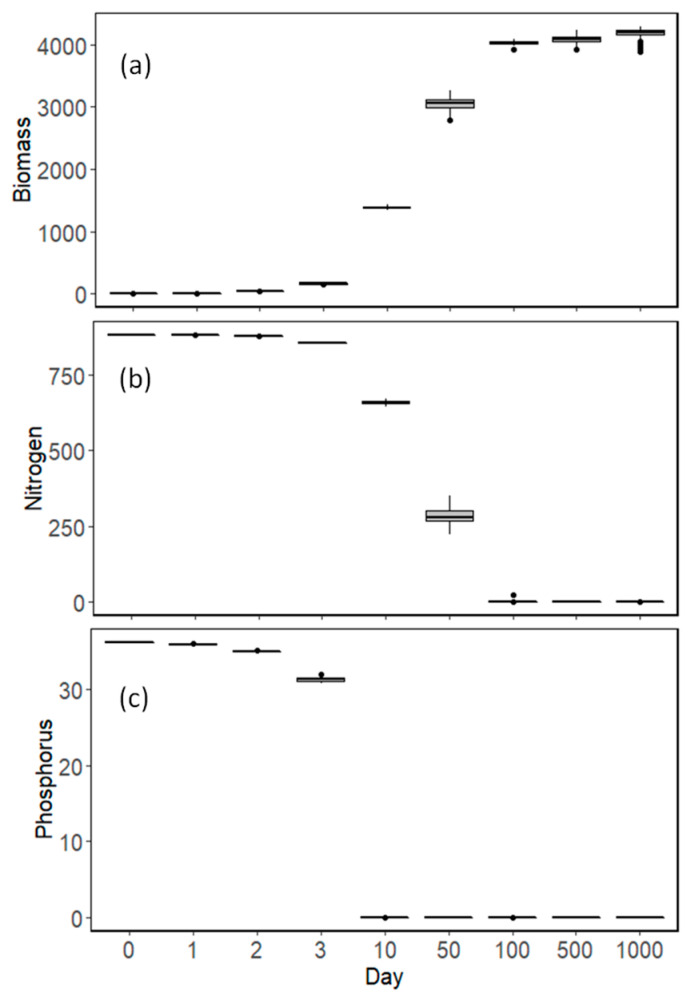
(**a**) Total biomass and nutrient concentrations; (**b**) nitrogen and (**c**) phosphorus during time succession in model simulations.

**Figure 6:**
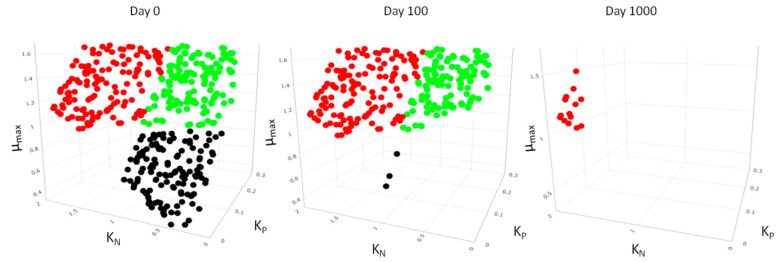
Three-dimensional diagram with the species life-history traits (μ_max_—maximum specific growth rate, K_N_-half saturation constant for nitrogen, K_P_-half saturation constant for phosphorus) in the model at day 0 (initial species pool), day 100 and day 1000 (end of simulation). Different colors are the three groups of species based on their competitive abilities (see methods for details).

Nutrient concentrations started to decline and reached their minimum fast, with the system being phosphorus-limited early in succession, whereas nitrogen concentrations took longer to decline (Figure 5). It was during this period of nitrogen depletion that total biomass increased further and reached its maximum when both nutrients reached their minimum values (Figure 5). During succession, the species that first went extinct were the slow-growing species (Figure 6), whereas, at the end of the simulation period, the species that survived and contributed the most to the total biomass were the ones with high growth rate and high competitive ability for phosphorus (i.e., low K_P_) (Figure 6).

## 4. Discussion

Overall, our study suggests that the BEF relationship in river benthic diatoms, although it could be regarded as positive, it seems to be a function of different factors. The main driver of the BEF seems to be geology, directly linked to nutrient availability, which, in turn, selects for specific functional traits. Furthermore, the maturity of the assemblage (i.e., time point during the succession of the biofilm) seems to be an important factor in the observed relationship, as suggested by the model simulations. This is the first attempt to generalize the BEF relationship in river benthic diatoms, using large-scale field observations and numerical modeling, and although the conclusion should be driven with caution, it offers valuable insight into these ecologically important assemblages.

The high variability among rivers regarding their environmental conditions (physico-chemical parameters, nutrient concentrations, drainage area) led to variable diatom assemblages and biomass production. Therefore, it was not surprising that the BEF relationship would also vary among rivers, greatly masking the effect of diversity on biomass in the whole dataset. However, when rivers were split into two groups based on the geology of the substrate, the two patterns were very clear: positive BEF in a siliceous substrate and no BEF in a calcareous substrate. The two substrate groups differed in nutrient concentrations, which were higher in sites with a siliceous substrate. This was expected for silica, as it originates from silicate rock weathering [38]. Regarding phosphorus, this pattern has already been shown in calcareous substrates, as phosphorus is being removed from the water column due to adsorption mechanisms on carbonate material [27,39,40]. Nitrogen was also lower in calcareous substrates, although this trend was not so pronounced. Although most of the sites were phosphorus-limited, there were some sites that were nitrogen limited, belonging, though, to both geological groups. This is consistent with previous studies in Greek rivers, suggesting that the limiting nutrient was site-dependent [26].

Nutrient concentrations largely affected diatom guilds. More specifically, motile species were more abundant in increased phosphate concentrations (and thus siliceous substrates), whereas low-profile species were more abundant in low phosphate concentrations (and thus calcareous substrates). This is in agreement with previous studies, where low-profile species showed a preference for low nutrient concentrations, whereas motile species abundance started to increase with increased nutrient concentration [13]. The fact that low-profile species (i.e., species that adhere strongly to the substrate) were more abundant in calcareous substrates could indicate that species in this functional group take advantage of the precipitated phosphorus. Furthermore, most of the low-profile species found in the study (especially *Achnanthidium* spp.) have a small size, are fast growers and tend to present high populations, increasing dominance [11]. 

The difference in the BEF relationship between the two substrates could be explained by the combination of nutrient concentrations and traits predominance and by the maturity of the biofilm. According to field data, at higher nutrient concentrations (siliceous substrate), the addition of species could increase biomass, suggesting that species do not occupy all available niches and new arriving species make use of available space, increasing biomass [41]. On the other hand, a stable BEF relationship suggests that the species present occupy all the available niches, consuming all the available resources, and the system has reached a saturated state, even from a few species [17,18]. Model results suggest that assemblages with a positive BEF could be at an early assembly process, whereas a stable relationship could be an indication of a later in succession, more mature assemblage. This is in agreement with previous modeling studies [17] for phytoplankton, using similar models but with different parameters regarding species’ life history traits. This could be an indication of a general trend in microalgae assemblages. 

Model outcomes suggest that the predominant species traits are related to fast growth and strong phosphorus competition. This is related to our field results, where phosphorus limitation was more predominant, and it would select species with low phosphorus requirements [35]. The selection for fast-growing species was also highly enforced by the penalty induced in slow-growing species, a rather simplistic function that selects for specific traits. The model applied in the present study followed many assumptions and generalizations and could not capture the complexity of a natural system. For example, nutrient inputs follow similar ratios as observed field nutrient concentrations but could be different in many cases, such as, for example, in highly polluted systems or when point-source pollution increases the concentration of a particular nutrient [42]. However, when comparing observational and model results, it is important to remember that it is not the absolute values that are being compared but rather the trends that could give indications on mechanisms underlying observed patterns. Therefore, we believe that our model results reinforce our field findings and assumptions on the time-dependent BEF observations. 

The lack of studies on the BEF relationship in benthic diatoms can be explained by a number of challenges and restrictions that it entails, some limitations of which were also apparent in the present study. Specifically, in rivers that are highly dynamic environments, biofilm assemblages can be highly affected by incidents such as heavy rains and floods and point source pollution that could make results evaluation harder. This was one of the reasons that sampling took place during summer, at low flow conditions, when there was a lower probability of heavy rain events, and we expected to collect a more mature biofilm. However, other stressors, such as pollution and desiccation, could be affecting our results [11]. Another limitation of benthic studies is the quantification of benthic concentrations and abundances and the overall sampling effort. In our study, we tried to eliminate this by scraping the biofilm of a defined surface and by using the same stone for both biomass measurement and diversity quantification. The use of chlorophyll a as a surrogate of ecosystem function is widespread in the literature, and it focuses on the biomass of primary producers of the biofilm and the general ecosystem state [43]. On the other hand, photosynthetic biofilm (i.e., phytobenthos) is a complex formation comprising many different groups of photosynthetic organisms apart from diatoms, including cyanobacteria. Therefore, different groups of species and pigments should be carefully considered in order to cover the full spectrum of the BEF relationship of the biofilm. Moreover, as water samples for the quantification of nutrients were from the water above the biofilm, and this differed from nutrient concentrations on the biofilm [44], the use of other ecosystem function metrics, such as the resource use efficiency (accounting for both biomass production and nutrient assimilation in cells, [6]), could not be directly related to our study. 

## 5. Conclusions

This was the first large-scale field study searching for a BEF relationship in benthic diatoms in rivers. Despite the limitations recognized in a field study on benthic microorganisms, it offers important insights into species’ contribution to biomass production. It highlights the importance of geology and nutrient concentrations on the form of BEF relationship and indicates species functional traits that could be responsible. The coupled modeling approach demonstrates the time-dependence of the BEF relationship during the succession of the biofilm formation and agrees with field observations on species functional traits. Further experimental work and application of different model scenarios could expand our knowledge and understanding of the ecosystem function of this ecologically important group of organisms. 

## Figures and Tables

**Table 1:** Slopes and their statistical significance for the equations. ln(biomass) = aS + b and ln(biomass) = aJ + b.

	Species Richness (S)		Evenness (J)	
Day	Slope (a)	*p*-Value	Slope (a)	*p*-Value
0	0.00286	***	31.01131	**
1	0.002282	***	2.686801	0.278
2	0.001267	*	0.447689	0.742
3	0.000544	0.569	−0.87027	0.445
10	0.00018	0.585	−0.05598	0.823
50	−0.00278	**	−1.21366	***
100	0.000241	0.170	−0.08981	**
500	0.000103	0.787	−0.24929	***
1000	−0.00434	**	−0.19818	***

Note: *** < 0.001, ** < 0.01, * < 0.05.

## Data Availability

Data are available on request.

## Appendix A

In Appendix A are figures and tables related to the methodology and detailed river results. All the available Figures and Tables are mentioned in the text.

**Table A1:** Coordinates and sampling dates of each site. Site numbers 1–5 correspond to sites from upstream to downstream.

River	Site	Latitude	Longitude	Sampling Date
Alfeios	1	37.36144	22.0947	2 July 2020
Alfeios	2	37.39018	22.08635	2 July 2020
Alfeios	3	37.47971	22.04998	2 July 2020
Alfeios	4	37.63421	21.64196	3 July 2020
Alfeios	5	37.64135	21.47642	3 July 2020
Arkadikos	1	37.26862	21.78473	21 July 2020
Arkadikos	2	37.2796	21.7412	21 July 2020
Arkadikos	3	37.28775	21.72578	21 July 2020
Arkadikos	4	37.29342	21.697	21 July 2020
Evrotas	1	37.17217	22.30336	1 July 2020
Evrotas	2	37.09295	22.42634	1 July 2020
Evrotas	3	37.06522	22.45116	1 July 2020
Evrotas	4	36.99387	22.51856	1 July 2020
Evrotas	5	36.97334	22.58183	1 July 2020
Fonias	1	40.45111	25.6258	22 August 2020
Fonias	2	40.4561	25.62369	22 August 2020
Fonias	3	40.45862	25.62405	22 August 2020
Fonias	4	40.48059	25.64669	22 August 2020
Fonias	5	40.49182	25.65536	22 August 2020
Lissos	1	41.13642	25.53514	7 September 2020
Lissos	2	41.02474	25.3223	7 September 2020
Lissos	3	41.0249	25.48959	7 September 2020
Lissos	4	41.0148	25.26305	7 September 2020
Mornos	1	38.59818	22.18833	10 July 2020
Mornos	2	38.51151	22.07488	10 July 2020
Mornos	3	38.50764	21.99866	10 July 2020
Mornos	4	38.50438	22.02188	10 July 2020
Mornos	5	38.38779	21.86056	10 July 2020
Neda	1	37.40079	21.9485	21 July 2020
Neda	2	37.4053	21.92258	21 July 2020
Neda	3	37.39259	21.84667	21 July 220
Neda	4	37.39526	21.72911	21 July 2020
Neda	5	37.38446	21.68998	21 July 2020
Nestos	1	41.41019	24.10549	4 September 2020
Nestos	2	41.26262	24.50997	4 September 2020
Nestos	3	41.17856	24.70111	4 September 2020
Nestos	4	41.08417	24.77134	5 September 2020
Nestos	5	40.99428	24.7438	5 September 2020
Spercheios	1	38.94828	21.94711	27 August 2020
Spercheios	2	38.94361	22.21083	27 August 2020
Spercheios	3	38.90667	22.28583	27 August 2020
Spercheios	4	38.89611	22.3225	27 August 2020
Spercheios	5	38.86722	22.36333	27 August 2020

**Table A2:** Model parameters.

Parameter	Explanation	Value/Range	Unit
i	species number	400	
j	number of resources	2	
μ_max_	maximum growth rate	0.3–1.7	d^−1^
K_P_	half-saturation constant for phosphorus	0.02–0.2	μM
K_N_	half-saturation constant for nitrogen	0.2–2	μM
c_P_	intracellular content for P	0.00397–0.055	μM
c_N_	intracellular content for N	0.055–0.244	μM
S_N_	input nitrogen concentration	882	μM
S_P_	input phosphorus concentration	36.2	μM
D	nutrient flushing rate	0.1	d^−1^
m_i_	species-specific flushing-induced mortality (D/μ_max_)	0.3–0.06	d^−1^
	total initial biomass	4 × 10^6^–5 × 10^6^	cells/cm^2^
	threshold abundance for a species’ survival	0.01 × 10^6^	cells/cm^2^
	range of initial abundance of each species	0.000009 × 10^6^–0.0225 × 10^6^	cells/cm^2^
